# Stride Segmentation during Free Walk Movements Using Multi-Dimensional Subsequence Dynamic Time Warping on Inertial Sensor Data

**DOI:** 10.3390/s150306419

**Published:** 2015-03-17

**Authors:** Jens Barth, Cäcilia Oberndorfer, Cristian Pasluosta, Samuel Schülein, Heiko Gassner, Samuel Reinfelder, Patrick Kugler, Dominik Schuldhaus, Jürgen Winkler, Jochen Klucken, Björn M. Eskofier

**Affiliations:** 1 ASTRUM IT GmbH, Am Wolfsmantel 2, Erlangen D-91058, Germany; E-Mails: jens.barth@astrum-it.de (J.B.); caecilia.oberndorfer@fau.de (C.O.); 2 Digital Sports Group, Pattern Recognition Lab, Department of Computer Science, Friedrich-Alexander University Erlangen-Nürnberg (FAU), Martensstraße 3, Erlangen D-91058, Germany; E-Mails: cristian.pasluosta@fau.de (C.P.); samuel.reinfelder@fau.de (S.R.); patrick.kugler@fau.de (P.K.); dominik.schuldhaus@fau.de (D.S.); 3 Department of Molecular Neurology, Universitätsklinikum Erlangen, Friedrich-Alexander University Erlangen-Nürnberg (FAU), Schwabachanlage 6, Erlangen D-91054, Germany; E-Mails: heiko.gassner@uk-erlangen.de (H.G.); juergen.winkler@uk-erlangen.de (J.W.); jochen.klucken@uk-erlangen.de (J.K.); 4 Geriatrics Centre Erlangen, Waldkrankenhaus St. Marien, Rathsberger Straße 57, Erlangen D-91054, Germany; E-Mail: samuel.schuelein@waldkrankenhaus.de

**Keywords:** inertial sensors, stride segmentation, accelerometer, gyroscope, dynamic time warping, free walk, gait analysis, Parkinson’s disease, geriatric patients, movement impairments

## Abstract

Changes in gait patterns provide important information about individuals’ health. To perform sensor based gait analysis, it is crucial to develop methodologies to automatically segment single strides from continuous movement sequences. In this study we developed an algorithm based on time-invariant template matching to isolate strides from inertial sensor signals. Shoe-mounted gyroscopes and accelerometers were used to record gait data from 40 elderly controls, 15 patients with Parkinson’s disease and 15 geriatric patients. Each stride was manually labeled from a straight 40 m walk test and from a video monitored free walk sequence. A multi-dimensional subsequence Dynamic Time Warping (msDTW) approach was used to search for patterns matching a pre-defined stride template constructed from 25 elderly controls. F-measure of 98% (recall 98%, precision 98%) for 40 m walk tests and of 97% (recall 97%, precision 97%) for free walk tests were obtained for the three groups. Compared to conventional peak detection methods up to 15% F-measure improvement was shown. The msDTW proved to be robust for segmenting strides from both standardized gait tests and free walks. This approach may serve as a platform for individualized stride segmentation during activities of daily living.

## 1. Introduction

The ability to automatically and robustly segment individual strides from gait sequences derived from inertial sensors is crucial for monitoring gait changes and answer specific clinical needs. The analysis of standardized gait examinations and free walking tests as well as home monitoring of disease-related patterns, risk-of-falls detection and other gait impairments becomes increasingly important for the elderly population, and during clinical assessment of movement disorders such as Parkinson’s disease [1].

Quantification of gait using inertial sensors attached to the foot has been used to answer clinical questions [1,2,3]. The development of such a system is commonly divided into three parts: (1) an accurate and robust segmentation of the gait sequences into individual strides; (2) a detection of specific gait events and a calculation of gait parameters; and (3) a comprehensive evaluation of these parameters in the context of the clinical questions. Here we focus on the first part and we propose an accurate segmentation of the measured sequence into single strides from straight walking on a flat surface.

Several algorithms for single stride analysis from accelerometer-derived data are based on peak detection methods [4,5,6]. Other studies [7,8,9] have used clearly defined signal characteristics like peaks or zero crossings in gyroscope and accelerometer data to determine gait events such as toe off, heel strike, and stance phase. Hundza *et al.* [10] used, for example, the gyroscope zero crossings to determine the initiation and termination of forward swing, defining toe off, and heel strike. These methods perform well if the sensor signals consist mainly of straight walking sequences. However, if strides need to be extracted from a free walk, as in the case of daily life activities at home, also sequences from e.g., climbing stairs might be miss-interpreted as strides.

Aminian *et al.* [11] used gyroscopes on the shin and thigh to identify strides during straight walking. This work was based on wavelet-based decomposition and threshold-based event detection. Gouwanda *et al.* [12] used the same approach but they decomposed the signal twice to identify the heel strike and toe off events directly. Khandelwal [13] combined wavelet transform with known gait features to extract gait events from accelerometer data during outdoor walking. Even though these wavelet-based methods were successfully implemented, they aimed at the identification of gait events from one sensor modality. None of them focused on an accurate identification of strides from free walking scenarios using combined sensor data.

A sequential stride phase classification algorithm is another possibility for segmenting strides from continuous walking. In this approach, instead of segmenting the complete stride sequence, single stride phases are detected and the correct order of these stride phases are used to define a stride. This methodology applied to accelerometer data was proposed by Han *et al.* [14]. Other groups used gyroscope-based systems to apply sequential stride phase identification algorithms [15]. They isolated the four gait phases stance, heel off, toe off, and heel strike by constructing threshold-based conditions using a-priori knowledge. Further, this approach was also implemented using a four-state left-right Hidden Markov Model [16,17]. A potential problem of this method is the detection of strides from abnormal gait (which was discussed by Mannini *et al.* [17]), where the signal patterns of single gait phases have a high variability.

Template-based cross-correlation methods have also been used for stride segmentation using either accelerometer [18] or gyroscope [19] data. The disadvantage of these methods is that the stride template has a fixed length and form, and thus it is not adaptable to different stride lengths and stride times. In our proposed algorithm, we use Dynamic Time Warping (DTW) [20] instead of cross-correlation. DTW allows the identification of patterns with different length and also matches signals non-linearly so that subparts of the template get stretched or shortened for an optimal fit by warping the template upon the target signal. DTW is a commonly used technique for computing the similarity between two time series.

DTW has also been used in the context of gait analysis. Segmentation of gait sequences extracted from video sequences using DTW enables comparison of test strides with reference strides [21]. Also, strides were first divided by filtering and peak detection, and then consecutively compared to reference strides via DTW for user identification [5]. DTW has mostly been used for comparing test strides to reference strides, but not for single stride segmentation. In this work, we implemented a special form of DTW called subsequence DTW [22] to identify single strides from a continuous signal.

Current inertial sensor based systems mostly use a combination of accelerometers and gyroscopes to record gait data [1,2,23,24,25]. However, the idea of automatically segmenting single strides from continuous and free walk sequences using combined data from accelerometers and gyroscopes is a current research question. Further, the subsequence DTW algorithm is a promising method for single stride segmentation, especially if the variability (even within individual persons) in length and amplitude of single gait sequences is considered [26]. A proof of concept has been published by our group were we used subsequence DTW to extract strides from sagittal plane gyroscope data only [27].

The purpose of this paper is to present a novel methodology for automatic single stride segmentation from standardized gait tests as well as continuous and free daily life walk movement sequences. This work focuses on the correct and robust separation between strides, which are similar to a given template, and other non-gait movements such as climbing stairs. Further analysis, including the computation of heel strike and toe off as well as stride length and stride times calculation, were presented in a separate publication [28]. We evaluate sensor modalities (either accelerometer or gyroscope) and we select optimum axes (and their combination) of combined gyroscope and accelerometer data for reliable stride segmentation. We finally demonstrate that the proposed method is applicable for clinically relevant applications and is consequently adaptable to different age groups, movement scenarios, and subject groups with movement disorders.

## 2. Methods

### 2.1. Subjects and Measurement Protocols

A total of 70 subjects were recorded for template generation and algorithm evaluation (Table 1). 25 elderly controls for template generation, 15 elderly controls and 15 patients with Parkinson’s disease (PD) for algorithm evaluation were recorded at the Movement Disorder Unit of the Department of Molecular Neurology, University Hospital Erlangen, Erlangen, Germany. Sensor data from 15 geriatric patients were acquired at the Geriatrics Centre, Waldkrankenhaus St. Marien, Erlangen, Germany. All subjects signed an informed consent form prior to the initiation of the study (ethical approval Re.-No. 4208, 21.04.2010, IRB, Medical Faculty, Friedrich-Alexander-University Erlangen-Nürnberg, Germany).

This study included two different protocols: (1) 40 m *walk:* Subjects walked four times 10 m at a comfortable self-selected speed in an obstacle-free environment. After each 10 m walk, subjects were instructed to turn 180 degrees in their transverse plane; (2) *free walk:* Subjects walked for two minutes around the hospital environment at a comfortable self-selected speed. This protocol was repeated two times. During the first two minute walk, subjects walked straight for 20 m, two times. The second two minute walk included climbing stairs for approximately 30 s. To mimic natural situations, between the 20 m walks and stair climbing, the participants performed sit to stand movements, walked straight, in curves, through doors, while these had to be opened and closed manually. The *free walk* protocol was videotaped for further sensor data labeling and an example video of a free walk is available in the supplementary information.

**Table 1:** Subject characteristics. m: male; f: female; SD: standard deviation; H & Y: Hoehn and Yahr [29]; UPDRS: Unified Parkinson’s Disease Rating Scale [30].

	Template	Controls		PD Patients		Geriatric Patients	
Test	40 m	40 m	free walk	40 m	free walk	40 m	free walk
Subjects	25	10	5	10	5	10	5
Strides	681	485	1286	496	1619	795	1249
Gender (m:f)	17:18	5:5	3:2	5:5	3:2	4:6	2:3
Age (±SD)	62.3 ± 11.6	64.0 ± 8.4	64.2 ± 10.0	63.8 ± 9.3	72.8 ± 6.3	81.0 ± 4.1	80.4 ± 5.9
Hoehn & Yahr (±SD)	-	-	-	1.7 ± 0.9	2.6 ± 0.5	-	-
UPDRS motor score (±SD)	-	-	-	12.7 ± 6.0	20.8 ± 6.1	-	-

### 2.2. Sensor System and Setup

Inertial measurement units (Shimmer 2R, Shimmer Sensing, Dublin, Ireland) were used to record acceleration and orientation data [31]. Each unit consists of a tri-axial accelerometer (MMA7260Q, Freescale Semiconductors, Austin, TX, USA) and a tri-axial gyroscope (500 series, InvenSense, Sunnyvale, CA, USA). The accelerometer measures linear accelerations in a range of ±6 g with a sensitivity of 200 mV/g. The gyroscope measures rotational velocities in a range of ±500 °/s with a sensitivity of ±2 mV/°/s. Data was recorded at a sampling rate of 102.4 Hz.

**Figure 1:**
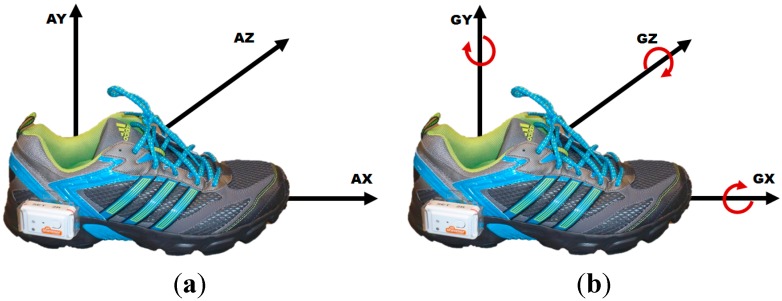
Shimmer sensor unit mounted with custom designed clip on the lateral side of a regular sport shoe. The directions of the sensor axes are shown accordingly to the sensor placement on the shoe. (**a**) Accelerometer; (**b**) Gyroscope.

The sensor units were mounted laterally to the heel of the subject’s right and left shoes to acquire data from both feet simultaneously (Figure 1). This sensor position was found to be optimal for stride analysis with inertial sensors [32]. In order to get comparable results, the same shoe model (adidas Duramo, with size customized to each subject) was used for all protocols. Data was collected via Bluetooth with custom software developed by ASTRUM IT GmbH (Erlangen, Germany).

### 2.3. Sensor Signals and Manual Data Labeling

**Figure 2:**
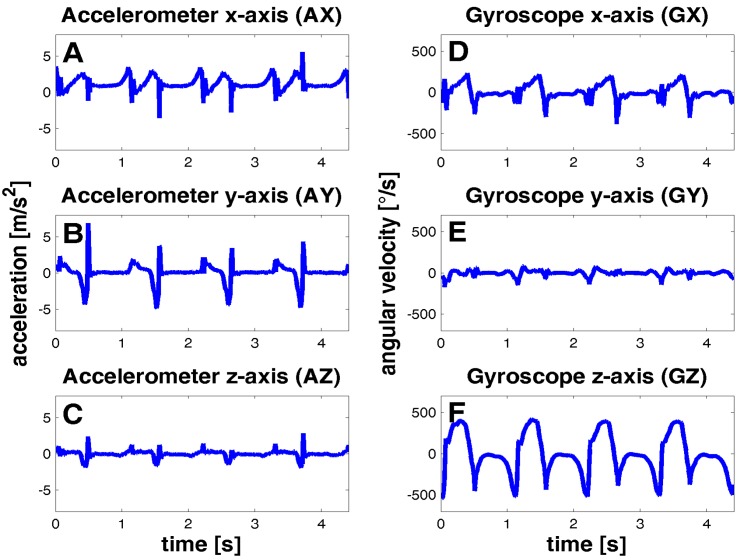
Typical sensor signals of four strides from a straight walk of an elderly control. Left column shows accelerometer signals from movements in (**A**) anterior-posterio; (**B**) inferior-superior and (**C**) lateral-medial direction. Right column shows gyroscope angular velocities of the rotations in (**D**) coronal, (**E**) transverse and (**F**) sagittal plane.

The accelerometer x-axis (AX) was defined in the anterior-posterior direction, the y-axis (AY) in the inferior-superior direction, and z-axis (AZ) in the lateral-medial direction (Figure 1). The angular velocities of gyroscope data were defined as rotations around x-axis (GX, coronal plane), y-axis (GY, transverse plane), and z-axis (GZ, sagittal plane). Signals were preprocessed and inverted to obtain equal sensor orientation for both feet (see Figure 2). The start and end point of each stride was labeled manually for template and gold standard generation based on the acquired information of the gyroscope.

Angular velocity in the sagittal plane (GZ in Figure 2) is commonly used for stride segmentation [10,17]. The positive peaks with the local maxima represent the swing phase, followed by the stance phase, which is the roll over movement from heel to toe. The negative peaks in GZ represent the change in the foot rotation during one stride and were used to define the stride start and end points. Stride start was set to the negative peak before swing phase and stride end to the negative peak at the end of the stance phase. The end of one stride might coincide with the start of the following stride for consecutive strides. For further analysis of continuous walking sequences, only strides from straight walking should be used and therefore only strides from regular and straight gait on flat surface were analyzed. Turning movements with more than 45 degrees per stride were excluded during the *free walk* protocol using videotaping to support manual data labeling.

### 2.4. Multi-Dimensional Subsequence Dynamic Time Warping for Stride Segmentation

The following section describes how different concepts of Dynamic Time Warping are combined for robust stride segmentation from several movement sequences.

#### 2.4.1. Principles of Multi-Dimensional Subsequence Dynamic Time Warping

**Figure 3:**
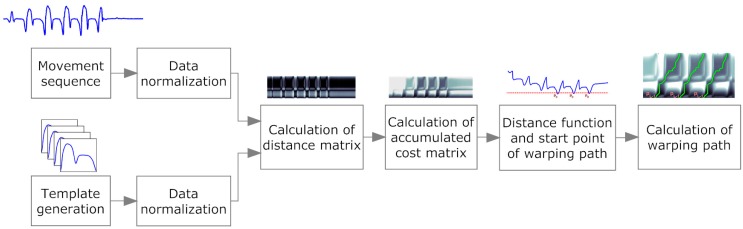
Illustration of the signal processing workflow for the msDTW algorithm, related to the organization of Subsection 2.4.2, Subsection 2.4.3, Subsection 2.4.4 and Subsection 2.4.5. The normalized movement sequence, from which the strides are extracted, and the normalized stride template were used to create a distance matrix. This matrix includes the distance between each sample of the stride template and each sample of the movement sequence. From this matrix, an accumulated cost matrix was generated that represents the costs of warping the stride template to the movement sequence. A path through this cost matrix with minimum costs represents a nonlinear warping of the template to a part of the movement sequence. Low costs indicate parts in the movement sequences that are very similar to the template. The start point of a warping path is identified in the distance function.

Dynamic Time Warping can be used to compare two signal sequences that may vary in time or speed [20]. This technique uses a similarity measure to represent the costs of warping a signal sequence to another. A variation of the Dynamic Time Warping algorithm is the subsequence Dynamic Time Warping (sDTW), used to find a subsequence of a continuous signal sequence similar to a given reference pattern [22]. In our study, multi-dimensional Dynamic Time Warping (mDTW) [33] was implemented to combine information from different sensor axes. We combined both techniques to a multi-dimensional subsequence Dynamic Time Warping (msDTW) to segment strides using information from different axes of the accelerometer and gyroscope at the same time. The complete workflow of the msDTW algorithm is illustrated in Figure 3.

#### 2.4.2. Continuous Movement Sequence

A continuous movement sequence $S_{raw}$ is defined as a signal from which strides are segmented. In the following the signals are described as of length *N* where $S_{raw}=(s_{0},\ldots ,s_{N-1})$. Each sample $s_{n}$ from $S_{raw}$ with n ϵ {0,…,N−1} consists of data from the accelerometer (AX, AY, AZ) and gyroscope (GX, GY, GZ).

#### 2.4.3. Template Generation

Stride template $T_{raw}$ of length M is defined as $T_{raw}=(t_{0},\ldots ,t_{M-1})$, where each sample $t_{m}$ with m ϵ {0,…,M−1} consists of data from the accelerometer and gyroscope sensor axes (AX, AY, AZ, GX, GY, GZ). Manually segmented strides using the GZ axis from the 40 m *walk* protocol were used to obtain $T_{raw}$. The dataset provided 681 strides from 25 different elderly controls with an average age of 62.3 years (Table 1).

**Figure 4:**
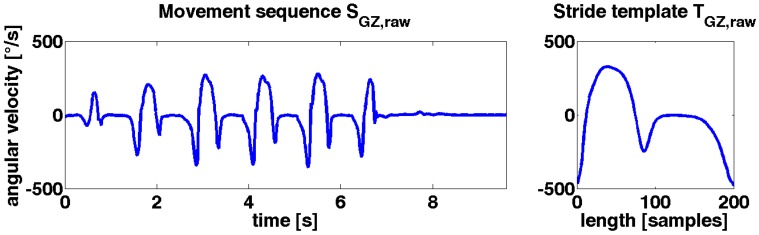
**Left**: the movement sequence $S_{Gz,raw}$ shows a typical angular velocity representation of a gait sequence in the sagittal plane (GZ); **Right**: the stride template $T_{Gz,raw}$ shows the template of the corresponding axis.

All 681 strides were averaged sample by sample to generate a template representative of a wide range of strides. All manually labeled strides were linearly interpolated to a length of 200 samples using the Matlab (Natick, MA, USA) function “interp1” previous to be averaged. This was done for each sensor axis of the gyroscope and accelerometer, separately. Figure 4 shows a representative sequence and template from the sensor axis GZ.

#### 2.4.4. Data Normalization

Each sensor axis was scaled to a range of [−1; 1] for the correct combination of the different sensor axes and data from accelerometer and gyroscope. This normalization was done by dividing the signals by the positive values of the sensor range, which were 6 g for the accelerometer data (A) and 500 °/s for gyroscope (G) data:

(1) $$S_{A,norm}=\frac{S_{A,raw}}{6\;g}$$

(2) $$T_{A,norm}=\frac{T_{A,raw}}{6\;g}$$

(3) $$S_{G,norm}=\frac{S_{G,raw}}{500\;{}^{\circ}/s}$$

(4) $$T_{G,norm}=\frac{T_{G,raw}}{500\;{}^{\circ}/s}$$

Even though only normalized data was used in further analysis, the index *norm* was omitted in the following text for simplicity.

#### 2.4.5. Calculation of Distance Matrix for Combined Sensor Data

**Figure 5:**
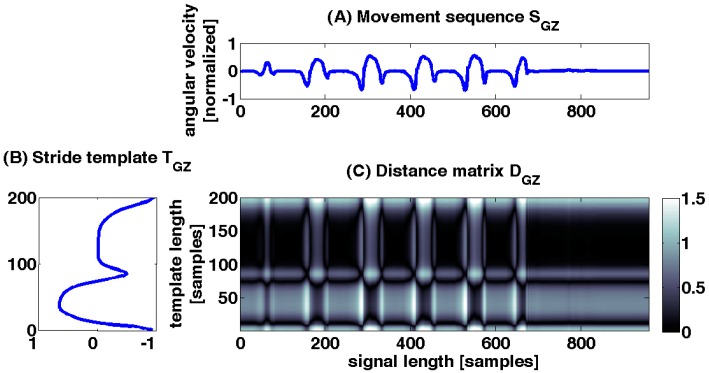
Distance matrix D_GZ_ is shown as an example for one sensor axis which is calculated from S_GZ_ and T_GZ_. Distance matrix was calculated from gyroscope angular velocity in sagittal plane (GZ). Deep black values in the distance matrix D_GZ_ show less distance and higher similarity between S_GZ_ and T_GZ_. White values signalize a high distance and consequently high costs. (**A**) movement sequence $S_{GZ}$; (**B**) stride template $T_{GZ}$; (**C**) distance matrix $D_{GZ}$;

A distance matrix *D* was constructed from the similarity measurements between the movement sequence *S* and the template *T*. *D* is a M×N matrix, where *M* is the length of the template *T* (rows) and *N* is the length of the movement sequence *S* (columns). Figure 5 shows an example for GZ. Each row in *D* represents the distance between one sample of the template *T* and the complete movement sequence *S*. The distance between every combination of samples from *T* and *S* can be calculated with any p-norm. In this work the Euclidian norm was used. The entries of *D* are defined as:

(5) $$D\left(m,n\right)=\sqrt{{\left(t_{m}-s_{n}\right)}^{2}}\;\forall \;m\;\epsilon \;\left\{0,\ldots ,M-1\right\},\;n\;\epsilon \;\left\{0,\ldots ,N-1\right\}$$

If samples $t_{m}$ and $s_{n}$ are similar, the local distance *d* is small and therefore has low costs. Contrary, a high value of *d* represents high costs and less similarity. The top row of *D* represents the distance between the end of the stride of the template *T* and the movement sequence *S*, while the bottom row represents the distance between the beginning of the stride of *T* and the sequence *S*.

A distance matrix *D* was constructed for each sensor axis of the accelerometer and the gyroscope and the distance matrices of all different single sensor axis were summed up to generate a new distance matrix. As an example, the combination of the sensor axes AY and GZ is given by:

(6) $$D_{AYGZ}\left(m,n\right)=\;D_{AY}\left(m,n\right)+D_{GZ}\left(m,n\right)\;\forall \;m\;\epsilon \;\left\{0,\ldots ,M-1\right\},\;n\;\epsilon \;\left\{0,\ldots ,N-1\right\}$$

Hence, the distance matrix could be treated as a regular distance matrix and further calculations were done as in a regular sDTW approach [22].

#### 2.4.6. Accumulated Cost Matrix and Warping Path

A path (p) with minimum costs from top to bottom of the distance matrix D represents a possible warping of stride template T to movement sequence S. To determine an optimal warping path p every possible path between T and S could be tested for lowest costs. However, this leads to a computational complexity that is exponential in the length M and N [22]. Therefore, a dynamic programming approach was used to reduce the complexity to O(MN). This was done by building an accumulated cost matrix C from the distance matrix D to search the optimal warping path p.

##### Calculation of Accumulated Cost Matrix

The accumulated cost matrix represents not only the distance between a template and a movement sequence, but also the accumulated costs of warping the template to parts of a movement sequence. An empty matrix with the same size as the distance matrix *D* was constructed to define the accumulated cost matrix *C*. The bottom row of *C* was filled with the bottom row of distance matrix *D*. This row is the source row to accumulate the costs of warping *T* to *S*.

(7) C(0,n)= D(0,n) ∀ n ϵ {0,…,N−1}

The first column of *C* was calculated by summing up the values from the bottom to the top of the first column of *D*. This was done for each element of the first column *C*(*m*,0):

(8) $$C\left(m,0\right)=\;\overset{m}{\underset{i=0}{\sum }}D\left(i,0\right)\;\forall \;m\;\epsilon \;\left\{0,\ldots ,M-1\right\}$$

The remaining elements *C*(*m*,*n*) were calculated by adding minimum cost elements of the accumulated cost matrix C to the respective elements from distance matrix *D* (from bottom to top). To calculate matrix element *C*(*m*,*n*) the matrix element *D*(*m*,*n*) was added to the minimal value of the three neighboring elements located to the left, below and left below of *C*(*m*,*n*). This accumulates the costs gradually from bottom to top and includes only minimal values for minimal costs:

(9) C(m,n)=min{C(m−1,n−1),C(m−1,n),C(m,n−1)}+ D(m,n) ∀ m ϵ {1,…,M−1}, n ϵ {1,…,N−1}

**Figure 6:**
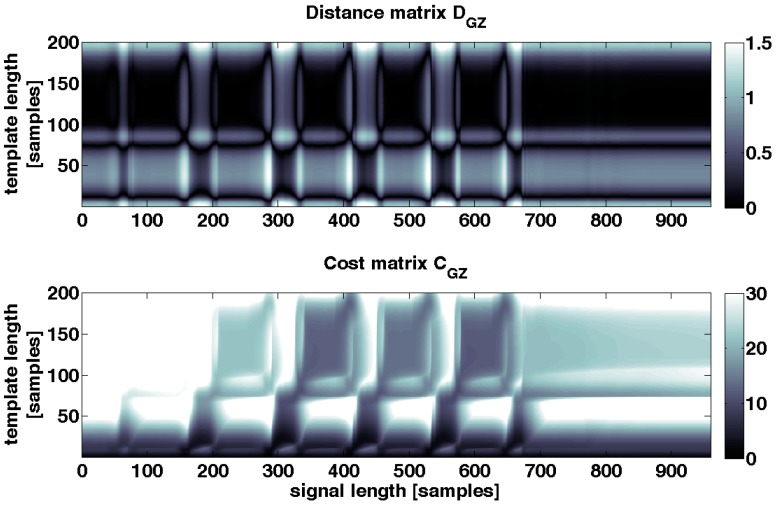
**Upper**: example distance matrix D_GZ_ for one axis, which is also shown in Figure 5; **Below**: cost matrix C_GZ_ which is calculated from D_GZ_. Deep black values in D_GZ_ and C_GZ_ show less distance and low costs between S_GZ_ and T_GZ_. White values signalize a high distance and high costs. A path of deep black values from top to bottom signalizes a good warping of stride template *T* to movement sequence *S*.

For example, the element *C*(4,5) is calculated by summing up *D*(4,5) = 5 and min{*C*(3,4) = 2, *C*(3,5) = 3, *C*(4,4) = 1}. As a result *C*(4,5) is equal to 6. The cost matrix C was then constructed using this procedure row by row. Therefore, the top row of the accumulated cost matrix C represents the accumulated costs of warping *T* to *S* (see Figure 6).

##### Distance Function and Starting Point of Warping Path

**Figure 7:**
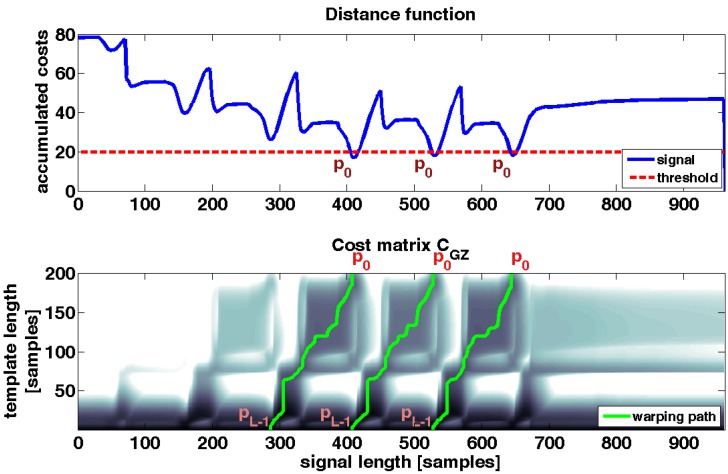
**Upper**: distance function Δ_*GZ*_ which is the top row of cost matrix C_GZ_. **Below**: cost matrix C_GZ_. In the plot of the distance function also the threshold and three local minima are illustrated. These minima are the start points of the warping paths *p*. The plot of the cost matrix C_GZ_ is overlaid with three warping paths *p* which correspond to three segmented strides in the movement sequence S_GZ_.

As a result of the calculation of the accumulated cost matrix, the top row of *C* represents the accumulated costs for warping stride template *T* to movement sequence *S* (Equations (7)–(9)). Because the cost matrix *C* was constructed from the distance matrix *D*, the top row represents also the stride end. The starting point of the warping path *p* is set to a local minimum of the top row of the cost matrix *C* and consequently to a minimum of the accumulated costs [22]. The top row of the cost matrix *C* is called distance function Δ and is visualized in Figure 7:

(10) Δ=C(M−1,n) ∀ n ϵ {0,…,N−1}

A local minimum from Δ was identified to select a starting point $p_{0}$ for the warping path. A criterion for the selection of local minima is the threshold τ (Figure 7). Each selected minimum, and consequently the accumulated cost of each selected end point of a stride, has to be smaller than threshold τ:

(11) $$\;p_{0}=\min\left\{\Delta \right\}=\min\left\{C\left(M-1,1\right),\ldots ,C\left(M-1,N-1\right)\right\}\;for\;\;p_{0}<\;\text{τ}$$

The threshold τ can be used as a regulating parameter for the sensitivity of the stride segmentation algorithm. The higher τ is the more minima are found, the more stride end points are identified and hence the more strides are detected (Figure 7). Given that the top row represents the accumulated costs, threshold τ limits the costs of warping template *T* to movement sequence *S*. Although the accumulated costs increases as the sampling rate of sequence *S* increases, the costs were still minimal since we search for local minima. Therefore, similarity is maintained across different sampling frequencies at these minimal points.

##### Calculation of the Warping Path

To find the start point of a stride, the path with the lowest costs from the top to bottom of the cost matrix needs to be calculated. The end point of this warping path *p* marks the starting point of a stride and the starting point of *p* defines the end point of a given stride.

The warping path *p* is of length *L* which can be different for each segmented stride.:

(12) $$p=\left(p_{0},\ldots ,p_{L-1}\right)\;\text{with}\;p_{l}=(m,n)\;\epsilon \;C\left(m,n\right)\;for\;l\;\epsilon \;[0,L-1]$$

A few boundary conditions need to be introduced for the complete stride to be correctly recognized in the cost matrix *C*. The following two conditions were necessary to ensure that the segmented stride maps the stride start (bottom row of *C*) and the stride end (top row of *C*) of the template *T*:

- Start of the warping path *p* is in top row of the cost matrix *C*:
  (13) $$p_{0}\;\epsilon \;\;\left\{C\left(M-1,0\right),\ldots ,C\left(M-1,N-1\right)\right\}$$
- End of the warping path *p* is in bottom row of the cost matrix *C*:
  (14) $$p_{L-1}\;\epsilon \;\left\{C\left(0,0\right),\ldots ,C\left(0,N-1\right)\right\}$$
- Next condition ensures that the warping path search is monotonically decreasing: Warping path *p* has to be a monotonic function where only neighboring elements are added. If new elements were added to *p*, at least one index must decrease. The maximum decrease of one index for a following element of the warping path is one:
  (15) $$p_{l+1}\;-p_{l}\;\epsilon \;\left\{\left(1,0\right),\left(0,1\right),\left(1,1\right)\right\}\;for\;l\;\epsilon \;\left[1,L-1\right]$$

The last condition was necessary because the starting point for the warping path search was the stride end, and the search for stride initiation needs to be performed earlier in time. Consequently, a warping path starts at the top row, is monotonically decreasing from top to bottom left and ends in the bottom row. With the first element in the bottom row the end of the warping path is reached.

#### 2.4.7. Constraints 

A warping path and, respectively, a stride was kept if the length of a warping path and hence of a stride is greater than 250 and less than 2000 ms. Additionally the time of an overlap of a given warping path must also be less than 100 ms for the stride to be recognized.

### 2.5. Peak Detection for Performance Comparison

A peak detection method was implemented to compare the performance of the msDTW based stride segmentation with respect to state-of-the-art methods. Here, only the gyroscope z-axis with the characteristic peak in the middle of the swing phase was used for detecting a stride [3,15,34]. The method was implemented as described in [3]. In this case, only one point in the stride and no stride borders were recognized. A peak was identified using the following conditions:

- The angular velocity must be greater than 150 °/s. Salarian *et al.* [3] used peaks larger than 50 °/s with shank mounted gyroscopes. In our study, the gyroscope threshold was increased since we used shoe-mounted gyroscopes, which produces higher angular velocities.
- The time distance to previous and following peaks must be greater than 250 ms. If multiple peaks within this region are detected, the highest amplitude is selected and the others are discarded [3].

### 2.6. Error Measurement

The error measurement criteria were chosen to adapt the threshold in order to minimize: (1) the number of missed strides and (2) the signal parts which are wrongly detected as strides. Here, a threshold adapted to not omit a stride (high precision), leads to the detection of arbitrary signal parts as strides. *Vice versa*, a threshold adapted to avoid arbitrary signal parts to be wrongly detected as strides (high recall), leads to missed strides. Therefore, F-measure was used as error measurement to optimize both issues equally [35].

#### 2.6.1. Precision

(16) $$precision=\frac{\sum^{\;}true\;positives}{\sum^{\;}detection\;positives}$$

The *detection positives* are the strides that were recognized as strides from the msDTW algorithm. The *true positives* are the strides that were recognized by the stride segmentation algorithm and also are labeled as strides in the gold standard data. The *precision* is then equal to one only if all the recognized strides were labeled in the gold standard data. Here, missing strides are not considered.

#### 2.6.2. Recall

(17) $$recall=\frac{\sum^{\;}true\;positives}{\sum^{\;}true\;positives+\sum^{\text{​}}false\;negatives}$$

*Recall* (also called sensitivity) considers the *false negatives*, which were the strides that were not recognized by the msDTW algorithm. This measurement is equal to one if no stride is missed. Here a stride which was wrongly detected is not considered.

#### 2.6.3. F-Measure

(18) $$F-measure=2\cdot \frac{precision\cdot \;recall}{precision+recall}$$

F-measure is the harmonic mean of precision and recall and it takes into account missing strides and wrongly detected strides equally.

The results from the msDTW algorithm were compared to the manually labeled stride borders and were marked as correctly segmented if the borders were within ±100 ms of the manually labeled stride borders, which is approximately 10% of stride time [17,36].

For the peak detection algorithm, a correct recognized stride was defined as a peak that lays in between the manually labeled stride borders of the gold standard data.

## 3. Experiments and Results

The evaluation of our proposed method was performed in three steps. First, the different sensor types and axes were evaluated using the data from the 40 m *walk*. Secondly, the axes with the best performance resulting from the previous step were combined and applied to the 40 m *walk* and *free walk* test for further analysis. Thirdly, the results of our methodology were compared to peak detection algorithm previously presented in the literature.

### 3.1. Separate Performance Evaluation of Accelerometer and Gyroscope 

To evaluate the performance of our algorithm on gyroscope and accelerometer data independently, the msDTW algorithm was implemented for each distinct axis and axes combination of both sensor modalities separately. To select the best sensor axis from each sensor modality, leave-one-subject-out cross-validation was performed using the data from the 40 m *walk*. For each evaluation, thresholds varying from 5 to 130 in increments of 5 were considered. For clarity, only F-measure results are listed in Table 2, presenting a general overview of the algorithm performance. The F-measures presented here are the mean values across cross-validation folds. Using only accelerometer data, best results for all tested groups were received from all three axes combined (AXAYAZ). Respectively, using gyroscope data alone, best results were received from the combination of GYGZ axis, also for all tested groups.

### 3.2. Stride Segmentation with msDTW and Combined Sensor Types

For a more in-depth evaluation, only the best performing axes of both sensor modalities were used (Table 2). The combination of accelerometer and gyroscope data was based on the best F-measures from the axes combination of single sensor types (AXAYAZ and GYGZ). Wrongly detected strides, missed strides, recall, precision and F-measure were also included in this evaluation. Results for all groups (controls, PD and geriatric patients) are shown in Tables S1 and S2 in the supplementary information. Table 3 shows an overview of the threshold, precision, recall and F-measures with average results across folds from the leave-one-subject-out cross-validation. Threshold values are plotted against precision, recall and F-measure values in supplementary information Figure S1.

**Table 2:** Stride segmentation results for accelerometer and gyroscope data from 40 m *walk* from msDTW given in F-measure values. Best results for each subject group are highlighted in bold numbers.

	X	Y	Z	XY	XZ	YZ	XYZ
	**Accelerometer Data**						
**Controls**	67%	53%	56%	80%	75%	79%	**85%**
**PD Patients**	73%	59%	32%	86%	77%	68%	**93%**
**Geriatric Patients**	47%	11%	44%	56%	38%	38%	**51%**
	**Gyroscope Data**						
**Controls**	80%	5%	95%	73%	97%	**97%**	96%
**PD Patients**	67%	5%	93%	39%	97%	**98%**	97%
**Geriatric Patients**	67%	4%	96%	48%	95%	**96%**	96%

The best results for 40 m *walk* were obtained using GYGZ with a F-measure of 98% for PD patients and 96% for geriatric patients. The combination of gyroscope and accelerometer achieved the best result for the control group with a F-measure of 98% (Table 3).

**Table 3:** Detailed results of 40 m *walk* and *free walk* from msDTW for best performing sensor axes of each sensor type separately (AXAYAZ and GYGZ) and for the combination of best axes from both sensor types (AXAYAZGYGZ). Overall best results for all groups are highlighted with bold numbers.

	40 M Walk				Free Walk			
	Threshold	Precision	Recall	F-Measure	Threshold	Precision	Recall	F-Measure
	Accelerometer Data, Combined AXAYAZ							
**Controls**	34.5	88%	90%	85%	33.3	90%	92%	90%
**PD Patients**	30.0	94%	94%	93%	34.5	82%	84%	81%
**Geriatric Patients**	35.0	60%	49%	51%	40.0	64%	62%	62%
	**Gyroscope Data, Combined GYGZ**							
**Controls**	34.8	96%	98%	97%	35.0	96%	97%	**96%**
**PD Patients**	30.0	98%	98%	**98%**	39.5	94%	97%	96%
**Geriatric Patients**	54.8	94%	98%	**96%**	50.0	94%	96%	**95%**
	**Combination of Accelerometer and Gyroscope Data AXAYAZGYGZ**							
**Controls**	70.0	97%	98%	**98%**	76.7	96%	97%	**96%**
**PD Patients**	70.0	98%	97%	97%	80.0	97%	97%	**97%**
**Geriatric Patients**	100.0	95%	93%	94%	104.0	82%	85%	83%

**Figure 8:**
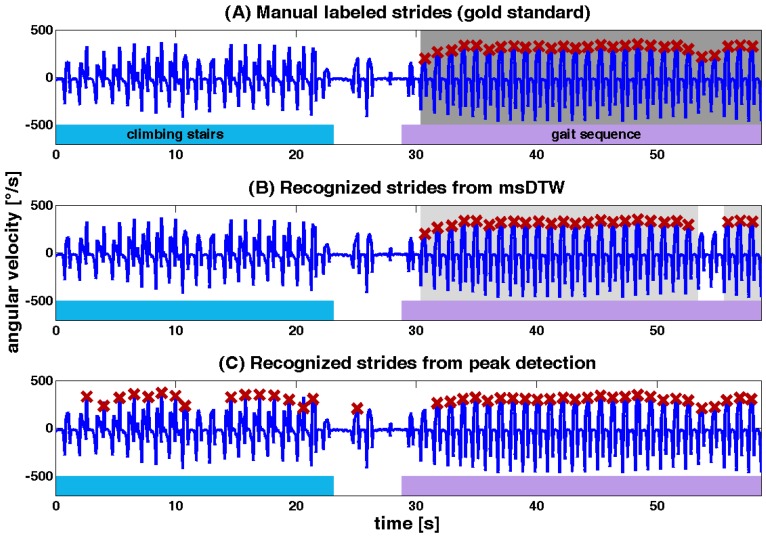
Excerpt of a typical gait signal from *free walk* of an elderly control. The subject climbed stairs for the first 20 s, which was followed by a transition to a straight walking episode. In the upper plot (**A**) manually labeled strides were marked with red crosses and the complete gait sequence in dark grey. The middle plot (**B**) shows the result from msDTW with recognized strides marked with red crosses and segmented gait sequence marked in grey. Lower plot (**C**) shows marked strides from peak detection algorithm. Only stride maxima were marked with red crosses, because peak detection only detects the peaks, not the complete gait sequence. Bottom plot also shows typical mistakes from the peak detection algorithm, where stair climbing strides by mistake were marked as strides.

The best results on *free walk* tests for controls and PD patients were obtained using the combination of accelerometer and gyroscope data AXAYAZGYGZ with a F-measure of 96% and 97%. For the group of geriatric patients the gyroscope GYGZ had the best performance with a F-measure of 95%. Plot B of Figure 8 shows a representative example for segmented strides with msDTW from a *free walk*.

### 3.3. Stride Segmentation Using Peak Detection

The stride segmentation using peak detection was based on GZ data only implemented as in Salarian *et al.* [3]. No parameter optimization was necessary. To evaluate the peak detection-based stride segmentation the same datasets as for msDTW evaluation were used.

The results for peak detection evaluation on 40 m walk data are presented in Table 4 (detailed results in supplementary information Tables S3 and S4). A maximum F-measure of 90% for geriatric patients and 86% for controls and PD patients were obtained using this methodology.

The results for peak detection evaluation on *free walk* data are presented in Table 4. A maximum F-measure of 90% for geriatric patients, 83% for PD patients and 81% for controls were obtained using this methodology. A representative example for segmented strides with peak detection from a *free walk* is shown in plot C of Figure 8.

**Table 4:** Detailed results of 4*0* m *walk* and *free walk* from peak detection algorithm based on data from GZ. The evaluated data corresponds to msDTW evaluated Section 3.2.

	40 M Walk			Free Walk		
	Precision	Recall	F-Measure	Precision	Recall	F-Measure
Controls	77%	99%	86%	68%	99%	81%
PD patients	76%	99%	86%	71%	100%	83%
Geriatric patients	84%	97%	90%	85%	95%	90%

## 4. Discussion

Sensor based gait analysis addressing clinical needs requires a three stages development concept: (1) a robust segmentation and identification of strides from sensor data; (2) the calculation of gait parameters from isolated strides; and (3) the selection and comprehensive analysis of individual stride parameters to answer relevant clinical question. This work addresses mainly the first part using the msDTW as a robust method to recognize and segment strides from inertial sensor data. The algorithm is able to correctly segment strides from healthy as well as altered gait, from both standardized gait tests such as the 40 m *walk* test, and from *free walk* sequences by simply adapting the threshold. In particular, the robust results from the *free walk* sequences support that the novel method is very suitable for home monitoring scenarios.

A comparison of stride segmentation resulting from accelerometer and gyroscope data showed that gyroscope based segmentation is required for high F-measures. Especially, the z-axis of the gyroscope (GZ), which represents the foot rotation in the sagittal plane, contributed mostly to the best results. If only straight walk tests were analyzed, good performance of stride segmentation from sagittal angular velocity signals has already been shown in the works of Hundza *et al.* [10] and Mannini *et al.* [16,17]. However, if turnings or other periodic movements like stair climbing were included in the walking sequence additional information would be required.

The aim of the study was to evaluate straight walking only. To improve the detection accuracy for this specific need, we added the information from GY to GZ, which improved the F-measure values by 2% and 5% for elderly controls and PD patients, respectively, during the 40 m *walk* tests. The GY axis represents the rotation around the superior-inferior axis and consequently had high amplitude on turning sequences. The template was constructed from strides while walking straightforward only. This allowed a precise rejection of turning movements, highly desirable for later analysis of isolated strides. However, to isolate turning movements for specific analysis, GY alone might be evaluated, or an appropriate template for turning movements can be defined.

Further evaluations showed that the combination of accelerometer and gyroscope data did not produce any major new insights, except in the case of *free walk* tests from the PD patients’ dataset, where the F-measure improved by 1%. However it is worthy to note that there is no study that has analyzed methods for stride segmentation using combined information from different sensor modalities simultaneously.

The F-measure from the geriatric patients’ dataset was not improved using the combination of accelerometer and gyroscope data. While our algorithm performs well on accelerometer data from controls and PD patients, the results based on the accelerometer from the geriatric patients dataset were non satisfactory (F-measure 51% for 40 m *walk* test and 62% for *free walk* test). These results together with the fact that for each experiment on the geriatric patient’s data a higher threshold delivers best results—a higher threshold means higher difference to the template—suggest that the template used in this study was not appropriate for this population. The template was defined from elderly controls data without gait impairments which were in average 18.7 years younger than the geriatric patients. Our group has demonstrated that the foot movement changes as the population gets older and that this plays an important role for identifying PD typical gait changes [1]. Especially the walking speed is lower in elderly, which may considerably influence the stride phases [37]. Future work may include an individualized template (age-matched, gender-matched…) for specific subject groups, which might improve these outcomes.

A potential issue of the proposed msDTW algorithm is that an optimum fitting threshold for each group and axes combination has to be identified. The focus of this study was to find the best threshold for one particular population and not to optimize the threshold in general. A possible solution to overcome this issue might be that a high threshold is used in general, which would lead to equal or better recall measurements at the expenses of a worse precision. This means that the number of wrongly detected strides will increase slightly. These wrongly detected strides may be rejected afterwards using post-processing methods with additional boundary conditions (e.g., temporal processing of a gait cycle, stride times, speed or turning angles).

One of the aims of this study was to determine which sensor axes combination performed best for which subject group. Our analysis showed that gyroscope GY combined with GZ axes performed very well for each group. The combination with accelerometer axes AXAYAZ increased the robustness of the results for controls and PD patients during the *free walk* tests. However, for geriatric patients the additional accelerometer axes did not improve the results for the same reasons discussed previously. The results showed that for the practical implementation of this algorithm the combination of GYGZ is highly recommended.

The developed algorithm was compared to an already published and well-described method from Salarian *et al.* [3]. As a difference we did not focus on gait event detection and we mounted the sensor directly on the shoe, not on the shank. The msDTW method showed 5% to 15% higher F-measures. Mainly, the precision was higher, whereas the recall was nearly perfect also for the peak detection method. The main reason therefore was that the peak detection algorithm interpreted each peak as a stride, which led to misinterpreted strides from other straight, non-gait movements such as climbing stairs. That means that nearly each stride was detected (high recall), but also numerous peaks were misinterpreted as strides (low precision). Thus, a peak detection approach performs well only if the movement sequence consists only of strides. In this study we focused on reliably extracting strides from completely free walk sequences. The results of this work showed that the msDTW algorithm is a suitable solution to this problem. Strides from *free walk* tests were recognized and segmented with high accuracy, while other movements were rejected (Figure 8).

A direct comparison with other studies that introduced stride segmentation algorithms was difficult because different error measurements were used in most of the studies. Another aspect that made the comparison challenging was the utilization of different datasets. Most of the groups analyzed data from gait sequences only, which included only signals from straight walking. Mannini *et al.* [17] e.g., used a Hidden Markov Model approach to identify strides from 2 min treadmill walking in different speeds and reached 94.9% sensitivity (also termed as recall) and 98.3% specificity. This approach has the advantage that also the gait phases where identified simultaneously. Compared to our results we reached a higher recall but our work did not include gait phase detection. Another difference is that Mannini *et al.* used data from treadmill walking not from *free walk* tests. Han *et al.* [14] recognized strides with an accuracy of 94% using accelerometers on both ankles during a continuous walking test of 6.5 m performed by using controls and 93% accuracy on PD patients. However, in the work of Han *et al.* it is not exactly defined how the accuracy measurements were calculated. The group of Hundza *et al.* [10] reported recognition rates of 100% from gyroscope data for a test where PD patients walked 25 m. This study implies that no strides were missed and no other movements were recognized as strides. Nonetheless, the data of Hundza *et al.* consists only of straight walking tests. No turning sequences, stair climbing or more than one gait initiation phase was included in these tests. This contrasts with our study where, especially in the *free walk* test, an arbitrary number of initiations, turning sequences and stair climbing strides were included, and the stride segmentation algorithm has to deal with much more challenging variations. Even in the 40 m *walk* tests there were three turnings and four gait initiations. Thus, even though our stride segmentation algorithm showed similar or even better results than previous works, the tests utilized here to evaluate our methodology was more complex by design.

## 5. Conclusions and Future Work

It was shown that msDTW is a robust method to segment strides from standardized gait tests and from *free walk* sequences. Compared to an implemented peak detection algorithm and to outcomes from previous studies similar results or better results were demonstrated. The consideration of data from *free walk* tests is novel in the field of stride recognition and also presented good results using our methodology. This approach may serve as the basis for individualized stride segmentation during activities of daily living, which may lead to an advanced gait analysis during daily life and open a novel window to a completely new direction for health and disease monitoring. To make results more amenable to comparison within the scientific community we published our data on www.activitynet.org and invite researchers to apply their methods on our dataset.

Future work includes the extension of this method to include the variation of different strides for template generation. Currently, the template was generated by averaging all strides. This was possible because the strides used in this study were very homogeneous. An optimized template may be generated by using DTW already for mapping the single strides to one template. Another method based on Hidden Markov Models used by Mannini *et al.* [17] may serve as a platform to account for different template conditions. Including information from both the right and left sensor simultaneously might improve the identification of possible existing alternating strides and should be considered for further research.

Finally, the inclusion of adaptive learning techniques are important for the practical implementation to select the best fitting template for each group and to find the optimum threshold during the algorithm is applied to individual datasets.

## Supplementary Materials

Supplementary materials can be accessed at: http://www.mdpi.com/1424-8220/15/3/6419/s1.
